# Effect of Er,Cr:YSGG Laser Irradiation with Radial Firing Tips on *Candida albicans* in Experimentally Infected Root Canals

**DOI:** 10.1155/2014/938245

**Published:** 2014-05-15

**Authors:** Leman Ozkan, Serap Cetiner, Tamer Sanlidag

**Affiliations:** ^1^Department of Pediatric Dentistry, Faculty of Dentistry, Near East University, Nicosia, Northern Cyprus, Mersin 10, Turkey; ^2^Department of Medical Microbiology, Celal Bayar University, Faculty of Medicine, 45000 Manisa, Turkey

## Abstract

*Aim*. To compare the disinfection effect of Er,Cr:YSGG laser using radial firing tips with NaOCI in root canals infected with *C. albicans* and to evaluate the irradiation effect on the dentinal surfaces. *Material and Methods*. In total seventy-six mandibular premolar teeth were used. In order to standardize the incubation and sterilization procedure, eight teeth were used. Sixty-eight of the root canals were incubated with *C. albicans* suspension for 72 hours. The specimens were divided into 5 experimental groups. Two groups were constituted as Group 1 was irradiated with 1.5 W laser (*n* = 8) and group 2, which was irradiated with 2 W laser (*n* = 8). Two more groups were formed as Group 3 (2 W laser (*n* = 25) and Group 4 NaOCI (5%) (*n* = 25). Group 5 (*n* = 2) did not receive any treatment. Mann-Whitney *U* and Kruskal-Wallis *H* tests were used to compare the different laser output powers. Wilcoxon Signed Ranks Test was used in order to compare the *Candida* cfu/ml levels according to treatment protocols (*P* < 0.05). *Results*. Both 1.5 W and 2 W laser resulted in a major reduction of *C. albicans* without a significant difference. The comparison of the dentin surfaces irradiated with Er,Cr:YSGG laser at two power settings resulted in similar morphological changes. However, NaOCI was found to be more effective in reduction of *C. albicans* than 2 W laser application. *Conclusion*. According to the results of the present study, the Er,Cr:YSGG laser with radial firing tips presented less antifungal effects on *C. albicans* in root canals of infected teeth than NaOCl solution.

## 1. Introduction

A number of factors have been identified as agents associated with failure of endodontic therapy, including intraradicular infections persisting in the apical part of the root canal, extraradicular infections, extruded root canal filling, or other materials that cause foreign body reactions and true cysts, especially those with a significant accumulation of cholesterol crystals [1, 2]. The most conclusive factor affecting the long-term outcome of endodontic treatment is persistence of infections in root canal system [3]. However the existence of accessory canals, anastomoses and fins, makes the complete elimination of debris, bacteria, and yeast harder because of limited penetration within the deeper layers of the dentin [4]. At least 300 different microbial species can be found in root canal infections of endodontic or periodontal origin [5]. Among these different species, yeasts have occasionally been found in primary root canal infections, but they seem to be more common in the root canals of obturated teeth in which the treatment has failed [6]. Waltimo et al. [7] reported that the majority of the recovered yeasts were* Candida* with* Candida albicans* (*C. albicans*) being the most predominant species.* C. albicans* can possibly be protected from the lethal action of endodontic medicaments because they are able to invade dentinal tubules to a variable extent [8]. In addition to the invading ability, it has been demonstrated that* C. albicans* are resistant to some medications commonly used in endodontics, such as calcium hydroxide [6]. Both features may help to explain why this particular fungal species has been associated with cases of persistent root canal infections [6].

Although several irrigants have been suggested in root canal disinfection over the years, sodium hypochlorite (NaOCl), at concentrations ranging from 0.5% to 5.25%, remains the most effective and most widely used [9]. However, improper usage at high concentrations can lead to complications during root canal irrigation, including accidental injection of NaOCl into the periapical tissues, air emphysema, and allergic reactions [10]. Additionally lasers have been suggested to assist endodontic treatments recently, because of their ability to remove the smear layer and to achieve efficient root canal system disinfection [11, 12]. The bactericidal effect of various laser systems in the root canal has been investigated, and most studies showed favorable results with regard to bacterial reduction [13–15]. Research on the use of one such laser system, Erbium, Cromium: Yttrium-Scandium-Gallium-Garnet (Er,Cr:YSGG), has primarily focused on the preparation of dental hard tissues and osseous and apical and periodontal surgery. Additionally erbium wavelengths can also be used in cleaning, shaping, and enlarging the root canal system and also is an efficient method to remove the smear layer [16–19].

Although favorable results have been achieved with the conventional forward emitting tips; the fiber tips still showed some room for improvement [20]. The beam geometry at the fiber tip resembles a narrow cone, delivering the largest part of the laser light straight towards the apex. Considering the fact that the diameter of the irradiated root canal is wider than the fiber diameter, a larger portion of the laser beam can be directed at the root canal walls just by tilting the fiber tip during the irradiation procedure. In order to prevent this, Er,Cr:YSGG laser underwent further improvement, and radial firing tips (RFT) has been developed that allow a more homogeneous irradiation of root canal walls. Modifications resulted with the ends of the RFT showing a conical outline with an angle of 60°. The outcome of this meant the laser light expanded to form a broad cone, facilitating an even and better coverage of the root canal wall [20].

Even though plenty of studies have investigated the bactericidal effects of Er,Cr:YSGG laser irradiation on microorganisms, no attempt has yet been made to evaluate the antifungal effects of Er,Cr:YSGG laser with the new generation fiber tips. Hence, it was considered worthwhile to compare the disinfection effect of Er,Cr:YSGG laser using radial firing tips with NaOCI in root canals infected with* C. albicans* and to evaluate the irradiation effect on the dentinal surfaces at different laser power settings together with the morphological alterations on dentinal surface using scanning electron microscopy (SEM).

## 2. Material and Methods

### 2.1. Sample Preparation

Seventy-six freshly extracted human mature permanent mandibular premolar teeth in orthodontic patients, with a single root canal and a closed apex, were used in this study; those having curved roots, more than one canal or any abnormal shape, were discarded. All patients gave written informed consent allowing the usage of their extracted teeth in our investigation.

The teeth were stored in saline solution following extraction. Bone, calculus, and soft tissues on the root surface were removed with curettes. The teeth were cut at 12 mm from the apex using a water-cooling diamond bur. The working length was set at 1 mm short of apical foramen. Root canals were prepared using the step-back technique with Ni-Ti K-files (K Files NITI FLEX, Dentsply, Germany) up to number 50 and Gates Glidden burs (numbers 2, 3, and 4) (JS Dental, Ridgefield, USA) which allowed entrance of the laser fiber to the apical area. The root canals were irrigated with 0.9% saline solution only; no EDTA (ethylenediaminetetraacetic acid) was applied during preparation. One investigator was assigned to perform all of the root canal preparations (LO).

After the preparation process, flowable composite resin (Clearfil Majesty Flow, Kuraray Medical Inc.) was used to seal the apex, whereas cotton pellets were used to seal the canal orifices followed by temporary filling material (3M ESPE, Cavit G). The roots were mounted vertically into Eppendorf tubes partially filled with acrylic resin. Once the acrylic resin sets, temporary filling material and cotton pellet were removed.

In order to standardize the incubation and sterilization procedure, eight teeth were selected randomly. The teeth were sterilized in order to check the outcome of the sterilization procedure. The teeth were put into a container with an indicator and autoclaved at 121°C for 15 minutes.

### 2.2. Microbial Inoculation

Following the procedure, initially four teeth were selected to standardize the inoculation process. A standard fungal culture of* C. albicans* (ATCC 90028) was obtained from Culture Collection*. C. albicans* was incubated for 72 hours on Saboraud Dextrose Agar (Oxoid Ltd., UK) at 37°C. 0.5 McFarland microbial suspension of* C. albicans* colonies was formed in Brain Heart Infusion Broth. Then 10 *μ*L of the microbial suspension was transferred into the canal lumen using sterile needles and incubated at 37°C for 72 hours. Following incubation, the microbial suspension was removed from the canal lumen and inoculated onto Saboraud Dextrose Agar to obtain the number of colony forming units. Following incubation, the number of* C. albicans* was found to be 100.000 cfu/mL, demonstrating their colonization.

### 2.3. Treatment Protocol

After standardization of the procedure, sixty-eight teeth were divided into 5 groups. All teeth were sterilized and inoculated according to the procedure above. In order to test the antifungal and the irradiation effect on the dentinal surfaces at different laser power settings, a power analysis (Power and Precision software, Biostat, Englewood, NJ, USA) was conducted to differentiate between two laser output power on sixteen teeth at a power of 0.8 (alpha = 0.05). Thus, the first step of the study was conducted using 16 teeth randomly selected and divided into two groups as follows.

**Group 1 (*n* = 8)**

The root canals received 1.5 W Er,Cr:YSGG laser treatment, output power of 0.6, 20 Hz, 25% water, and 35% air.

**Group 2 (*n* = 8)**

The root canals received 2 W Er,Cr:YSGG laser treatment, output power of 0.45, 20 Hz, 25% water, and 35% air.

Then, two more groups were constituted in order to compare the disinfection effect of Er,Cr:YSGG laser with NaOCI according to the law of large numbers.

**Group 3 (*n* = 25)**

The root canals received 2 W Er,Cr:YSGG laser treatment, output power of 0.45, 20 Hz, 25% water, and 35% air.

**Group 4 (*n* = 25)**

5% NaOCI was used as a root canal irrigation solution. The samples were irrigated with 2 mL of NaOCl for 1 minute.

**Group 5 (*n* = 2)**

The root canals did not receive any treatment and served as the control group.

For laser irradiation, each sample was treated with one lasing cycle, which consisted of four irradiations of ten seconds each with 5 second intervals in between, where the fiber tip was inserted all the way down into the apex. Then, the laser was activated and the root canal was irradiated from apical to cervical region with helicoidal movements. One investigator was assigned to perform all of the irradiations (LO).

### 2.4. Microbiological Evaluation

Following laser treatment, the root canals were rinsed with 1 mL of saline solution (0.9%). From each root canal 10 *μ*L of remnant saline solution was removed using a sterile needle and cultured on Saboraud Dextrose Agar at 37°C for 72 hours in order to obtain the number of fungal colonies (cfu/mL). The colonies were then counted and the total number of fungus (colony forming units per mL of the extraction fluid) was assessed.

### 2.5. Scanning Electron Microscopy (SEM)

In the first part of scanning electron microscopy (JSM 6400, JEOL, Tokyo, Japan) evaluation, we compared two different power settings (1.5 W and 2 W) to evaluate the morphologic changes induced by laser irradiation (6 teeth in total, 3 samples from Group 1 and 3 samples from Group 2). We observed that there were no significant differences between the two laser energy settings. In the second section of the SEM analysis, in order to achieve greater antifungal effect, setting of 2 W power output was used against NaOCl irrigation (8 teeth in total, 4 samples from Group 3 and 4 samples from Group 4). The preparation of the samples involved making longitudinally grooves on the buccal and lingual surfaces of the root with a diamond bur. These grooves were used as guidance to split the roots into two pieces. The samples were gold sputtered and mounted on metal stubs for examination. Apical, middle, and coronal thirds of the samples were evaluated separately with the aid of scanning electron microscopy.

### 2.6. Statistics

Statistical analysis was carried out using the SPSS 12.0.1 (SPSS, Chicago, ILL) software program. Mann-Whitney *U* and Kruskal-Wallis *H* tests were used to compare the different laser output powers. Wilcoxon Signed Ranks Test was used in order to compare the* candida* cfu/mL levels according to treatment protocols. A *P* value of less than or equal to 0.05 was considered statistically significant.

## 3. Results

### 3.1. Comparison of Laser Power Settings

The disinfection effects of the Er,Cr:YSGG laser on the infected root canals of the extracted human teeth are reported in Table 1. At 1.5 W laser power setting 67.7% elimination of* C. albicans* was achieved whereas at 2 W laser power setting, 75.4% elimination of* C. albicans* was obtained. Significant difference was found for both laser power settings before and after irradiation (*P* < 0.05). However, there was no difference between 2 W and 1.5 W laser output powers according to* C. albicans* elimination (*P* = 0.083).

**Figure 1:**
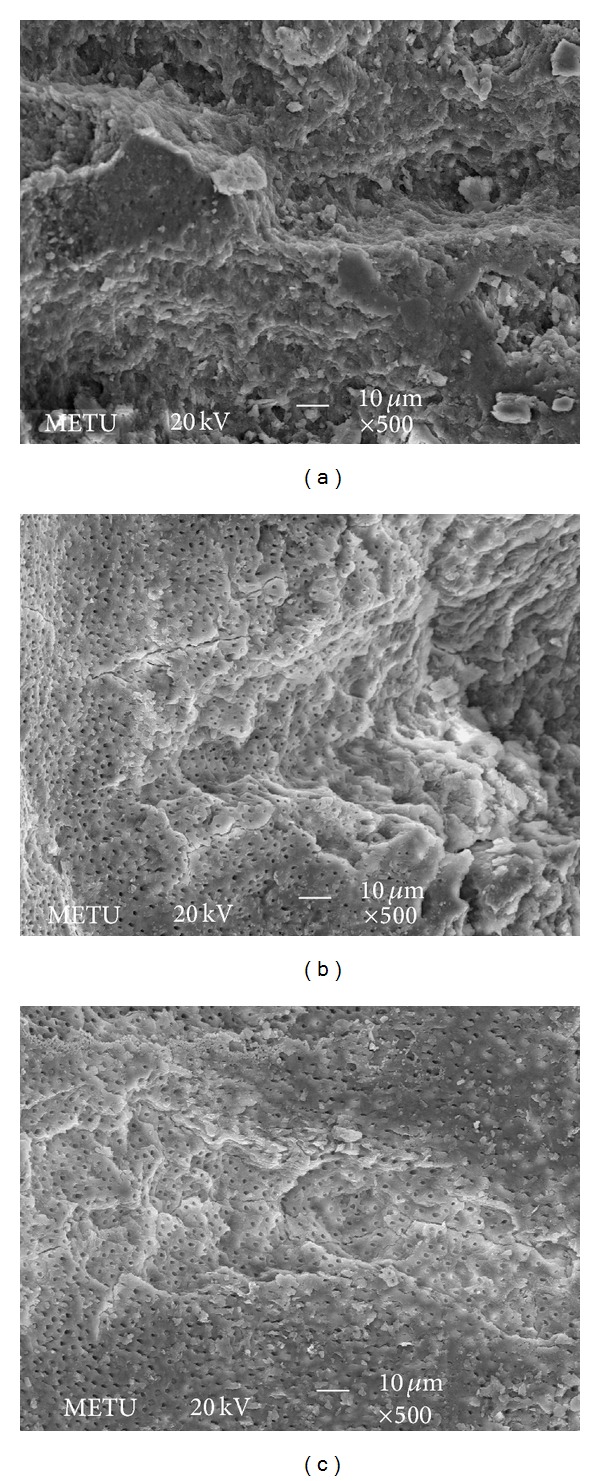
SEM photos showing the samples treated with 1.5 W Er,Cr:YSGG laser: (a) apical third, (b) middle third, and (c) coronal third; magnification ×500.

**Figure 2:**
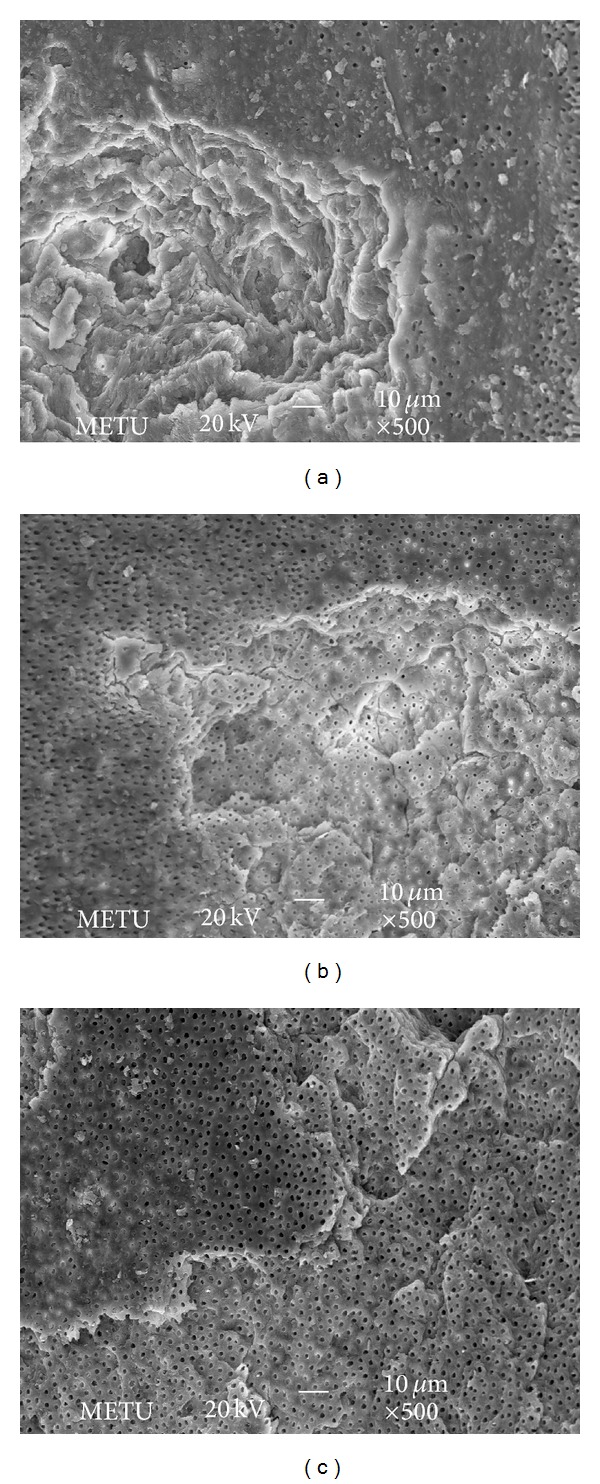
SEM photos showing the samples treated with 2 W Er,Cr:YSGG laser: (a) apical third, (b) middle third, and (c) coronal third; magnification ×500.

Moreover, the comparison of the dentin surfaces irradiated with Er,Cr:YSGG laser both at 1.5 W and 2 W resulted in similar morphological changes. SEM images showed irregular irradiation for both power settings. The fiber optic used in the helicoidal technique promoted irregular irradiation and thus crack formation over the root surface (Figures 1 and 2). Exposed dentinal tubules that were free of debris and smear layer were clearly visible at both wavelengths and there was no sign of either carbonization or melting. The irradiated surfaces showed irregular areas, which looked like cracks and fissures (Figures 1 and 2).

### 3.2. Comparison of Laser and NaOCI

The comparison of the Er,Cr:YSGG laser and NaOCI on the infected root canals reported in Table 2. At 2 W laser power setting 79.1% elimination of* C. albicans* was achieved whereas 100% elimination of* C. albicans* was obtained using NaOCI. Significant difference was found before and after laser irradiation (*P* = 0.019). There was also statistical significance for* C. albicans* elimination using NaOCI. All fungal microorganisms were eliminated after NaOCI irrigation (*P* = 0.001).

**Figure 3:**
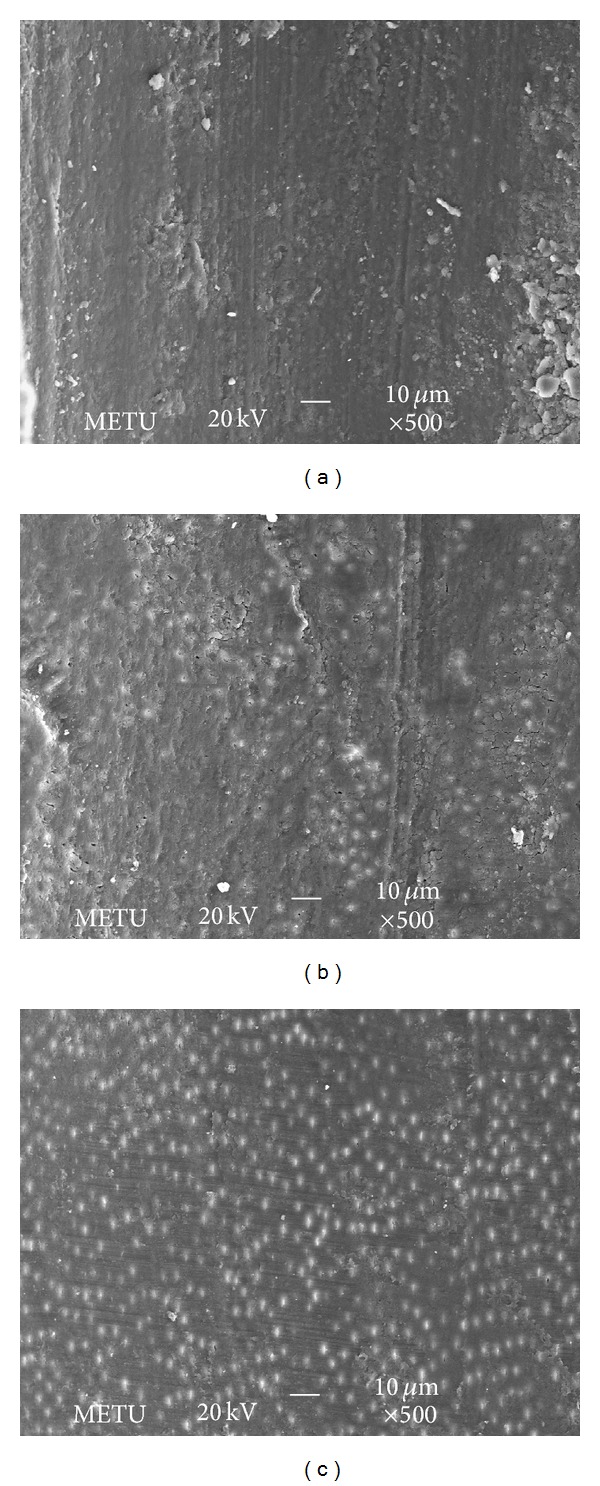
SEM photos showing the samples irrigated with NaOCl: (a) apical third, (b) middle third, and (c) coronal third; magnification ×500.

Examination of the specimen with SEM after NaOCI treatment revealed that most of the dentinal wall was covered by a smear layer; only few open tubules were visible (Figure 3). The smear layer was getting thicker from coronal to apical third of the teeth. Moreover the smear layer in NaOCI irrigated teeth was excessive to those with laser irradiated ones.

## 4. Discussion

There is presently considerable research activity on new methods and materials used for instrumentation, irrigation, disinfection, and filling of the root canal space to achieve complete elimination of root canal infection and to prevent reinfection.

Previous studies on this subject indicated a wide range of results. Berutti et al. [21] stated that the pathogenic microorganisms are able to penetrate the root dentin up to a depth of more than 1 mm, whereas disinfection solutions only reach a depth of 100 *μ*m. Laser irradiation has the potential to aid in endodontic treatment [16], not only because of its improved removal of debris and smear layer, but also because of its ability to provide greater accessibility to formerly unreachable parts of the tubular network [22]. The high penetration depth of the laser light into the dentinal tissue seems to be the most logical explanation for the successful bactericidal effectiveness of different laser wavelengths [20]. Due to the adjustable penetration depth of the laser irradiation, lasers can result in better access to complex regions of the root canal system, compared with rinsing solutions [23].

All lasers in the Erbium family have been shown to reduce bacteria in the root canal system [24]. Several studies were conducted using laser irradiation with and without NaOCI and their effect was investigated particularly on* Escherichia coli* (*E. coli*) or* Enterococcus faecalis* (*E. faecalis*). Schoop et al. [25] used Er,Cr:YSGG laser with power settings of 1 W and 1.5 W on two test bacteria—*E. coli* or* E. faecalis*. They concluded that Er,Cr:YSGG laser was effective in eliminating the microorganisms. Wang et al. [17] compared 1 W/1.5 W Er,Cr:YSGG laser, neodymium-doped yttrium aluminum garnet laser (Nd:YAG), and 2.5% NaOCI. They have shown that both laser systems have significant bactericidal effects. However Nd:YAG was more effective than Er,Cr:YSGG laser. Franzen et al. [26] investigated Er,Cr:YSGG laser at a pulse energy of 3.13 mJ and found a significant bacterial reduction up to a dentin thickness of 500 *μ*m. Eldeniz et al. [16] compared NaOCI and Er,Cr:YSGG irradiation in contaminated root canals; they concluded that Er,Cr:YSGG laser reduced but did not eradicate all bacteria. Recently Yavari et al. [27] stated that 2 W and 3 W powers of Er,Cr:YSGG had antibacterial effects on* E. faecalis* in root canals. Arnabat et al. [18] indicated that 2 W laser for 60 seconds was effective as NaOCI 5% for reduction bacterial colony. However, only very limited studies was conducted on laser application and its effect on* C. albicans*. Onay et al. [28] used Er,Cr:YSGG laser at power settings of 1 W and 0.75 W in conjunction with and without NaOCl. They reported a higher fungal reduction (92%) with combination treatment of NaOCl and 1 W laser.

In this study, we evaluated the effectiveness of Er,Cr:YSGG laser irradiation through a radial firing tip with a diameter of 200 *μ*m on* C. albicans* and also determined the morphological alterations on dentinal surface at different power settings using a scanning electron microscope.

As the study of Yamazaki et al. [29] revealed carbonization and crack formation all over the root canal surface at irradiation above 2 W, we conducted this study at power settings of 1.5 and 2 W. As a result, 67.7% microbiological elimination was achieved at a lower power setting of 1.5 W, whereas better results were obtained at a higher power setting of 2 W (75.4%) without a statistical difference. This result is in line with Onay et al. [28], but the reduction rate was lower than their study. This can be attributed to their combination therapy of NaOCl and 1 W laser usage.

Moreover, we compared 2 W laser treatment with 5% NaOCI for fungal reduction, where complete elimination of* C. albicans *with 5% NaOCI was obtained. NaOCI performed better results for disinfection of* C. albicans *from root canals. However, the Er,Cr:YSGG laser with radial firing tips demonstrated a considerable effect on yeast reduction within infected root canal. The different laser power settings had similar cleaning effects for root canals infected with* C. albicans*.

Recently, new studies have been conducted using the “new” radial firing tip as in our study. This upgrade led to more uniform exposure of the whole dentinal surface due to the conical shape of the fiber tip, which allowed laser light to be emitted in the form of a broad cone with an angle of about 60° [20].

Gordon et al. [30] used the radial firing tip in Er,Cr:YSGG laser and compared with NaOCI in order to eliminate* E. faecalis*. They concluded that 120 seconds application of laser had greater disinfection effect than with NaOCI alone. Schoop et al. [20] also used Er,Cr:YSGG with radial firing tips (0.6 W and 0.9 W) on two test bacteria—*E. coli* or* E. faecalis*. Through their findings they stated that the radial firing tip is a suitable tool for elimination, especially with a higher diameter of 300 *μ*m or 400 *μ*m, allowing a higher energy output ranging around 1.5 W. Scoop et al. [25] also tested the same equipment at 1 W and 1.5 W and found similar results which indicated reduction of number of bacterial colonies. Recently Martins et al. [31] conducted an in vivo study using Er,Cr:YSGG with the radial firing tips and compared this with 3% NaOCI and interim calcium hydroxide paste. They concluded that these tips can be used with Er,Cr:YSGG laser in endodontic treatment with less restriction and adverse effects than irrigation solutions. Although no study was conducted with radial firing tips on* C. albicans,* our results were in accordance with the findings showing that radial firing tips made significant bacterial reduction.

The effects of NaOCl and Er,Cr:YSGG laser on smear layer and the morphological alterations on dentinal surfaces were compared in our study. We did not use EDTA in the current study since it has the potential to remove the smear layer after root canal preparation [32, 33]. Rather than using EDTA, the removal potential of Er,Cr:YSGG laser on smear layer was evaluated. It has been demonstrated that mechanical instrumentation creates a smear layer of calcified detritus that adheres to the dentinal surface. In order to decrease the amount of smear layer on root canal surfaces, irrigants and antiseptic agents are used, enhancing the effect of hand instrumentation and improving the efficiency of the sodium hypochlorite. However, studies have shown that a combination of NaOCl and EDTA remove the smear layer only partially [12, 32, 33]. In addition to this, Sen et al. [34] investigated the antifungal effects of 0.12% chlorhexidine (CHX) and 1% and 5% NaOCl on* C. albicans* and concluded that both the smear layer and biofilms of* C. albicans* delayed or stopped the antifungal capacity of NaOCl and CHX. Thus, we attempted to evaluate the effect of Er,CR:YSGG laser on smear layer.

Through SEM, we found that the Er,Cr:YSGG laser at power settings of 1.5 and 2 W removes the smear layer and debris from the root canal walls and opens up the dentinal tubule orifices. This should help the practitioners seal the root canal tightly. The irradiated surfaces showed irregular areas, which looked like cracks and there were no signs of carbonization or melting in our SEM images. Our findings coincide with the results of Yamazaki et al. [29], which reported carbonization and crack formation all over the root canal surfaces when irradiated with more than 2 W. Schoop et al. [25] observed both damaged surfaces on the periluminal dentin together with melting and crystallization of the dentinal surfaces at power settings of 1 and 1.5 W. It was thought to be due to the laser being used without the water-air delivery system. The findings from other studies have indicated that after irradiation without water spray, carbonization is seen in enamel and dentin, associated with an irregular structure with many microholes [25, 29, 35]. As a result, throughout our study we applied laser irradiation with water-air delivery system which showed no melting or crystallization as in Schoop et al.'s study [25]. Similar to our results Silva et al. [19] showed that root canal surfaces were free of smear layer and dentinal tubules were opened at power settings of 1.5 and 2 W. There were no signs of melting and carbonization, only cracks and fissures were observed as in our study. It can be concluded that using Er,Cr:YSGG laser with water-air delivery system decreases the possibility of melting and carbonization.

Er,Cr:YSGG laser device that emits a laser beam having a wavelength of 2.78 *μ*m has a mechanism of laser energy interaction with water at the tissue interface and has therefore been termed a hydrokinetic system (HKS). It has been speculated that the mechanism of cutting involved absorption of laser energy by fine water droplets, resulting in a violent yet controlled microexpansion that included strong mechanical forces on targeted tissue surface [35]. This resulted in hydrokinetic forces that produce mechanical separation of the calcified tissue surfaces causing quick and clean tissue removal [36]. Highly magnified observation by SEM analysis in our study revealed concave and convex surfaces and cracks that were thought to be caused by microablation. Our results support the former hypothesis of laser ablation mechanism of HKS.

As a limitation of the current study, there were differences between the sample sizes of the experimental groups. The first two groups (*n* = 8) were used as a prior pilot study for confirming the reliability of the efficiency of different output powers. As a result of this, it has been decided to use 2 W laser irradiation in root canal disinfection. After standardization of this procedure, the remaining fifty-two teeth were divided into 3 groups as Group 3 (*n* = 25), Group 4 (*n* = 25), and Group 5 (*n* = 2).

The other possible limitation may be the lack of SEM analysis before the root canal preparation and laser irradiation. It would be useful and interesting to have the images of intact root canal surfaces in order to compare with the alterations after root canal preparation and laser irradiation. Further studies should be done in order to test these limitations.

## 5. Conclusions

According to the results of the present study, the Er,Cr:YSGG laser with radial firing tips presented less antifungal effects on* C. albicans* in root canals of infected teeth than NaOCl solution. NaOCl solution inhibited the growth of* C. albicans *and effectively disinfected all root canals. When the Er,Cr:YSGG laser's smear layer removal effect is considered, combination usage of lower concentrations of NaOCl and Er,Cr:YSGG laser should be evaluated in follow-up studies.

## Figures and Tables

**Table 1:** Mean values of microbial counts *(C. albicans)* before and after laser irradiation in different output powers.

Group	*X* ± SD (initial)	*X* ± SD (final)	*P* value	Fungal reduction mean (%)
2 W laser (*n* = 8)	83750 ± 31139	21250 ± 2474	0.010*	75.4%
1.5 W Laser (*n* = 8)	75000 ± 26726	25000 ± 7071	0.011*	67.7%

*P < 0.05; X: mean value; SD: standard deviation.

**Table 2:** Mean values of microbial counts *(C. albicans)* before/after laser irradiation and NaOCI irrigation.

Group	*X* ± SD (initial)	*X* ± SD (final)	*P* value	Fungal reduction mean (%)
2 W laser (*n* = 25)	96000 ± 13843	20000 ± 2670	0.019*	79.17%
NaOCI (*n* = 25)	90400 ± 18814	0 ± 0	0.001*	100%

*P < 0.05; *X*: mean value; SD: standard deviation.

## References

[1] Rôças IN, Siqueira JF, Aboim MCR, Rosado AS (2004). Denaturing gradient gel electrophoresis analysis of bacterial communities associated with failed endodontic treatment. *Oral Surgery, Oral Medicine, Oral Pathology, Oral Radiology and Endodontology*.

[2] Siqueira JF (2001). Aetiology of root canal treatment failure: why well-treated teeth can fail. *International Endodontic Journal*.

[3] Chávez de Paz LE, Dahlén G, Molander A, Möller Å, Bergenholtz G (2003). Bacteria recovered from teeth with apical periodontitis after antimicrobial endodontic treatment. *International Endodontic Journal*.

[4] Byström A, Sundqvist G (1981). Bacteriologic evaluation of the efficacy of mechanical root canal instrumentation in endodontic therapy. *Scandinavian Journal of Dental Research*.

[5] Sundqvist G (1994). Taxonomy, ecology, and pathogenicity of the root canal flora. *Oral Surgery, Oral Medicine, Oral Pathology*.

[6] Siqueira JF, Sen BH (2004). Fungi in endodontic infections. *Oral Surgery, Oral Medicine, Oral Pathology, Oral Radiology, and Endodontics*.

[7] Waltimo TMT, Sirén EK, Torkko HLK, Olsen I, Haapasalo MPP (1997). Fungi in therapy-resistant apical periodontitis. *International Endodontic Journal*.

[8] Haapasalo HK, Sirén EK, Waltimo TMT, Ørstavik D, Haapasalo MPP (2000). Inactivation of local root canal medicaments by dentine: an in vitro study. *International Endodontic Journal*.

[9] Siqueira Júnior JF, Rôças IN (2008). Clinical implications and microbiology of bacterial persistence after treatment procedures. *Journal of Endodontics*.

[10] Hülsmann M, Hahn W (2000). Complications during root canal irrigation—literature review and case reports.. *International Endodontic Journal*.

[11] George R, Meyers IA, Walsh LJ (2008). Laser activation of endodontic irrigants with improved conical laser fiber tips for removing smear layer in the apical third of the root canal. *Journal of Endodontics*.

[12] Takeda FH, Harashima T, Kimura Y, Matsumoto K (1998). Comparative study about the removal of smear layer by three types of laser devices. *Journal of Clinical Laser Medicine and Surgery*.

[13] Lee MT, Bird PS, Walsh LJ (2004). Photo-activated disinfection of the root canal: a new role for lasers in endodontics. *Australian Endodontic Journal*.

[14] Stabholz A, Sahar-Helft S, Moshonov J (2004). Lasers in endodontics. *Dental Clinics of North America*.

[15] Schoop U, Kluger W, Moritz A, Nedjelik N, Georgopoulos A, Sperr W (2004). Bactericidal effect of different laser systems in the deep layers of dentin. *Lasers in Surgery and Medicine*.

[16] Eldeniz AU, Ozer F, Hadimli HH, Erganis O (2007). Bactericidal efficacy of Er,Cr:YSGG laser irradiation against Enterococcus *faecalis* compared with NaOCl irrigation: an ex vivo pilot study. *International Endodontic Journal*.

[17] Wang Q-Q, Zhang C-F, Yin X-Z (2007). Evaluation of the bactericidal effect of Er,Cr:YSGG, and Nd:YAG lasers in experimentally infected root canals. *Journal of Endodontics*.

[18] Arnabat J, Escribano C, Fenosa A (2010). Bactericidal activity of erbium, chromium:yttrium-scandium-gallium-garnet laser in root canals. *Lasers in Medical Science*.

[19] Silva ACB, Guglielmi C, Meneguzzo DT, Aranha ACC, Bombana AC, de Paula Eduardo C (2010). Analysis of permeability and morphology of root canal dentin after Er,Cr:YSGG laser irradiation. *Photomedicine and laser surgery*.

[20] Schoop U, Barylyak A, Goharkhay K (2009). The impact of an erbium, chromium:yttrium-scandium-gallium-garnet laser with radial-firing tips on endodontic treatment. *Lasers in Medical Science*.

[21] Berutti E, Marini R, Angeretti A (1997). Penetration ability of different irrigants into dentinal tubules. *Journal of Endodontics*.

[22] Klinke T, Klimm W, Gutknecht N (1997). Antibacterial effects of Nd:YAG laser irradiation within root canal dentin. *Journal of Clinical Laser Medicine and Surgery*.

[23] Odor TM, Chandler NP, Watson TF, Pitt Ford TR, McDonald F (1999). Laser light transmission in teeth: a study of the patterns in different species. *International Endodontic Journal*.

[24] Perin FM, França SC, Silva-Sousa YTC (2004). Evaluation of the antimicrobial effect of Er:YAG laser irradiation versus 1% sodium hypochlorite irrigation for root canal disinfection. *Australian Endodontic Journal*.

[25] Schoop U, Goharkhay K, Klimscha J (2007). The use of the erbium, chromium:yttriumscandium-gallium-garnet laser in endodontic treatment: the results of an in vitro study. *Journal of the American Dental Association*.

[26] Franzen R, Esteves-Oliveira M, Meister J (2009). Decontamination of deep dentin by means of erbium, chromium:yttrium- scandium-gallium-garnet laser irradiation. *Lasers in Medical Science*.

[27] Yavari HR, Rahimi S, Shahi S (2010). Effect of Er, Cr: YSGG laser irradiation on enterococcus faecalis in infected root canals. *Photomedicine and Laser Surgery*.

[28] Onay EO, Alikaya C, Seker E (2010). Evaluation of antifungal efficacy of erbium, chromium: yttrium-scandium- gallium-garnet laser against Candida albicans. *Photomedicine and Laser Surgery*.

[29] Yamazaki R, Goya C, Yu D-G, Kimura Y, Matsumoto K (2001). Effects of erbium,chromium:YSGG laser irradiation on root canal walls: a scanning electron microscopic and thermographic study. *Journal of Endodontics*.

[30] Gordon W, Atabakhsh VA, Meza F (2007). The antimicrobial efficacy of the erbium, chromium:yttrium-scandium- gallium-garnet laser with radial emitting tips on root canal dentin walls infected with Enterococcus faecalis. *Journal of the American Dental Association*.

[31] Martins MR, Carvalho MF, Vaz IP, Capelas JA, Martins MA, Gutknecht N (2013). Efficacy of Er, Cr:YSGG laser with endodontical radial firing tips on the outcome of endodontic treatment: blind randomized controlled clinical trial with six-month evaluation. *Lasers in Medical Science*.

[32] O’Connell MS, Morgan LA, Beeler WJ, Baumgartner JC (2000). A comparative study of smear layer removal using different salts of EDTA. *Journal of Endodontics*.

[33] Andrabi SM, Kumar A, Mishra SK, Tewari RK, Alam S, Siddiqui S (2013). Effect of manual dynamic activation on smear layer removal efficacy of ethylenediaminetetraacetic acid and SmearClear: an in vitro scanning electron microscopic study. *Australian Endodontic Journal*.

[34] Sen BH, Safavi KE, Spångberg LS (1999). Antifungal effects of sodium hypochlorite and chlorhexidine in root canals. *Journal of Endodontics*.

[35] Kimura Y, Yu D-G, Kinoshita J-I (2001). Effects of erbium, chromium:YSGG laser irradiation on root surface: morphological and atomic analytical studies. *Journal of Clinical Laser Medicine and Surgery*.

[36] Rizoiu I, Kohanghadosh F, Kimmel AI, Eversole LR (1998). Pulpal thermal responses to an erbium,chromium:YSGG pulsed laser hydrokinetic system. *Oral Surgery, Oral Medicine, Oral Pathology, Oral Radiology, and Endodontics*.

